# Collagen structures of demineralized bone paper direct mineral metabolism

**DOI:** 10.1093/jbmrpl/ziae080

**Published:** 2024-06-17

**Authors:** Hyejin Yoon, Yongkuk Park, Jun-Goo Kwak, Jungwoo Lee

**Affiliations:** Department of Biochemistry and Molecular Biology, University of Massachusetts, Amherst, MA 01003, United States; Molecular and Cellular Biology Graduate Program, University of Massachusetts, Amherst, MA 01003, United States; Department of Chemical Engineering, University of Massachusetts, Amherst, MA 01003, United States; Molecular and Cellular Biology Graduate Program, University of Massachusetts, Amherst, MA 01003, United States; Molecular and Cellular Biology Graduate Program, University of Massachusetts, Amherst, MA 01003, United States; Department of Chemical Engineering, University of Massachusetts, Amherst, MA 01003, United States; Department of Biomedical Engineering, University of Massachusetts, Amherst, MA 01003, United States

**Keywords:** bone matrix, collagen, matrix mineralization, osteoblasts, osteoclasts

## Abstract

Bone is a dynamic mineralized tissue that undergoes continuous turnover throughout life. While the general mechanism of bone mineral metabolism is documented, the role of underlying collagen structures in regulating osteoblastic mineral deposition and osteoclastic mineral resorption remains an active research area, partly due to the lack of biomaterial platforms supporting accurate and analytical investigation. The recently introduced osteoid-inspired demineralized bone paper (DBP), prepared by 20-μm thin sectioning of demineralized bovine compact bone, holds promise in addressing this challenge as it preserves the intrinsic bony collagen structure and retains semi-transparency. Here, we report on the impact of collagen structures on modulating osteoblast and osteoclast-driven bone mineral metabolism using vertical and transversal DBPs that exhibit a uniaxially aligned and a concentric ring collagen structure, respectively. Translucent DBP reveals these collagen structures and facilitates longitudinal tracking of mineral deposition and resorption under brightfield microscopy for at least 3 wk. Genetically labeled primary osteogenic cells allow fluorescent monitoring of these cellular processes. Osteoblasts adhere and proliferate following the underlying collagen structures of DBPs. Osteoblastic mineral deposition is significantly higher in vertical DBP than in transversal DBP. Spatiotemporal analysis reveals notably more osteoblast adhesion and faster mineral deposition in vascular regions than in bone regions. Subsequent osteoclastic resorption follows these mineralized collagen structures, directing distinct trench and pit-type resorption patterns. In vertical DBP, trench-type resorption occurs at an 80% frequency, whereas transversal DBP shows 35% trench-type and 65% pit-type resorption. Our studies substantiate the importance of collagen structures in regulating mineral metabolism by osteogenic cells. DBP is expected to serve as an enabling biomaterial platform for studying various aspects of cellular and extracellular bone remodeling biology.

## Graphical Abstract

**Graphical Abstract:**
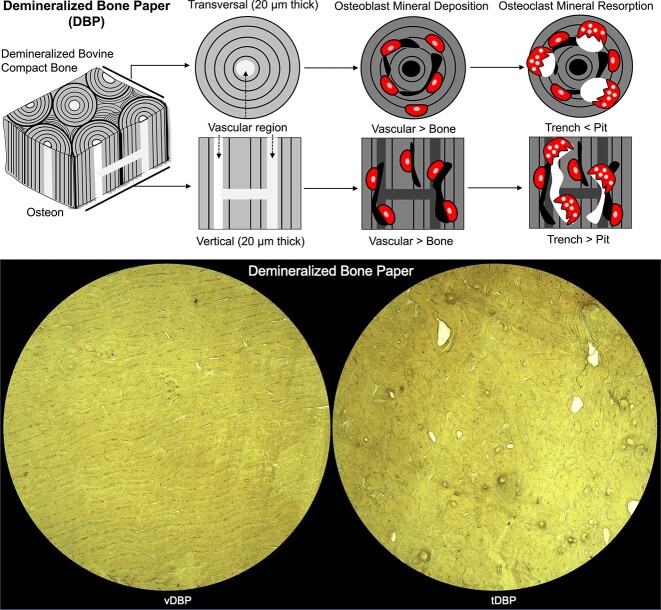

## Introduction

Mineralized bone formation and resorption are essential for maintaining mechanical structure and mineral homeostasis throughout life. Osteoblastic bone formation begins with the secretion of procollagens, which are processed and assembled into collagen fibrils. These collagen fibrils interweave and cross-link to form the rudimentary bone structure known as osteoid.^1^ Osteoblasts then mineralize the osteoid by releasing biomolecules essential for the nucleation and growth of minerals on collagen fibrils. When osteoid is fully mineralized, osteoblasts become resting-state lining cells until the bone-forming cycle resumes. During bone resorption, BM monocytes merge into multinucleated osteoclasts on the bone surface, breaking down the mineralized collagen matrix.^2^^,^^3^ While these cellular and molecular processes have been documented, the effect of the underlying collagen structure in regulating mineral metabolism remains an active area of research, partly due to the inherent challenges in studying osteogenic cellular processes and the related mineral metabolism on the inner surface of bone cavities in living animals.

Mechanistic investigations of bone mineral metabolism have been primarily conducted in vitro, which can be divided into acellular and cellular approaches. Acellular mineralization involves a collagen matrix and simulated body fluid (SBF), which mimics the ionic composition and pH of blood plasma.^4^^,^^5^ This approach has provided valuable insights into the mechanisms underlying biomineralization. For example, it has been discovered that nano-scale gaps within collagen fibrils create a favorable local energy environment for the nucleation of hydroxyapatite crystals. Subsequent transport of calcium and phosphate ions induces the growth of minerals along the collagen fibers, leading to interfibrillar mineralization.^6-8^ Nevertheless, it remains unknown how the natural organization of the collagen matrix directs long-range macro-scale acellular mineralization and how this compares cellular mineralization.

Osteoblastic mineralization has been studied using various collagen-coated biomaterials with osteogenic medium.^9^ These studies highlight the importance of the collagen matrix in modulating osteoblast adhesion, growth, and mineralization.^10^^,^^11^ However, most studies use collagen derived from soft tissues, such as skin and tendon, which may have different osteo-inductive and osteo-conductive properties than bony collagen. These extracted collagen fibers are reconstituted via surface adhesion, gelation, and freeze-drying to reflect native bone extracellular matrix complexity.^12^^,^^13^ Nevertheless, these approaches are limited in replicating the multiscale hierarchical structure and organization of collagen in natural bone.

Demineralized bone matrix, which preserves the natural collagen structure of bone, represents an opportunity for studying in-vivo–relevant mineralization. However, it has been mainly used as morselized particles for clinical bone tissue regeneration.^14^ Several studies have identified the importance of physical properties such as particle sizes in regulating mineralization capacity,^15^ but this approach has seldom been applied to mechanistic studies. Besides, the irregular 3D shape of demineralized bone particles limits detailed microscopic observation of mineralized collagen formation by osteoblasts.

Osteoclastic mineral resorption has been commonly studied on bone discs, 100-500 μm thick bovine compact bone, as they closely reproduce osteoclast morphology and mineral resorption process.^16^^,^^17^ Murine BM mononuclear cells (BMMs) and human CD14^+^ cells differentiate into multinucleated osteoclasts with macrophage-colony stimulating factor and receptor activator of nuclear factor kappa beta (RANKL).^18^^,^^19^ Microscopic observation identifies 2 distinct resorption types. When osteoclasts dissolve the bone matrix while migrating, it creates a narrow, elongated groove known as a trench. When osteoclasts resorb bone stationary, they generate round depressions known as pits.^20^ These resorption patterns provide insights into the activity and function of osteoclasts in bone remodeling and could serve as diagnostic markers for assessing bone quality. Despite the potential influence of underlying mineralized collagen structures on osteoclast resorption patterns, a detailed investigation into this relationship has yet to be conducted.

Here, we report how the collagen structure of bone modulates osteoblast and osteoclast-mediated mineral metabolism by exploiting recently introduced demineralized bone paper (DBP), an osteoid-inspired biomaterial. DBP is prepared by sectioning the demineralized bovine compact bone matrix into 20-μm thin slices. DBP preserves the structural integrity and organization of the bony collagen matrix. Semi-transparent DBP allows monitoring of fluorescent osteogenic cellular processes and mineral metabolism under brightfield microscopy. Previously, DBP demonstrated functional and analytical recapitulation of in-vivo–relevant osteoblast and osteoclast processes.^21^^,^^22^ One unique feature of DBP is its distinct collagen structure, which depends on the sectioning direction. Vertically sliced DBP (vDBP) has a uniaxially aligned collagen structure, whereas transversally sectioned DBP (tDBP) has a concentric ring collagen structure. By exploiting these unique features of DBP, we first examine the influence of DBP collagen structures on acellular mineralization in SBF, leveraging the increasing darkness of mineralized collagen matrix under brightfield microscopy. A randomly deposited collagen matrix is used as a control. Next, we investigate osteoblast adhesion, proliferation, and mineralization as a function of DBP collagen structures. Primary osteoblasts retrieved from DsRed reporter mice allow longitudinal fluorescent monitoring of cellular processes on DBPs. The deposited mineral structure and composition are analyzed by scanning electron microscopy with energy dispense X-ray spectroscopy. Finally, we determine the impact of mineralized collagen structures in directing osteoclast mineral resorption. Osteoblasts on DBP exposed to vitamin D3 (VD3) and prostaglandin E2 (PGE2) induce differentiation of BMMs into multinucleate osteoclasts. The resulting osteoclastic mineral resorption is monitored by exploiting locally increased brightness of demineralized collagen matrix under brightfield imaging. Comparative spatiotemporal analysis of mineral deposition and resorption between vDBP and tDBP substantiates the importance of collagen structures in regulating osteoblast and osteoclast-mediated mineral metabolism. We envision that DBP will be a valuable biomaterial platform for in-depth and analytical investigation of bone mineral metabolism.

## Materials and methods

All chemicals and materials were purchased from Sigma-Aldrich or Fisher Scientific unless specified. All animal procedures were approved by the Institutional Animal Care and Use Committee of the University of Massachusetts Amherst. Experiments with and handling of mice were conducted in accordance with federal, state, and local guidelines.

### Demineralization of bovine cortical bone

Frozen bovine femurs were obtained from local slaughterhouses or grocery stores and cut into ~5 cm in length with a bone saw. Inner marrow and outer muscular connective tissues were manually removed. Residual fats were dissolved with a 1:1 chloroform and methanol mixture for 1 d while replacing the solution in 12 h. Cleaned shaft bones were immersed in a 1.2 N hydrochloric acid solution to dissolve minerals. The container holding the bones was placed into a cyclic hydrostatic pressure chamber to accelerate demineralization. Compressed air (4 bars) was applied to the chamber for 10 s on–off cycle (0.1 Hz). After the first 24 h of operation, the outer bone layer was removed with a razor blade to increase the surface area of the bone for demineralization. Next, the hydrochloric acid solution was replaced daily for 5-7 d until fully demineralized. The demineralization process was checked by bending and the complete demineralization was confirmed by X-ray scanning (IVIS Spectrum-CT). The demineralized bone pieces were then placed in deionized (DI) water overnight to remove residual hydrochloric acid. Finally, demineralized bone pieces were either processed immediately or saved at −20 °C after vacuum sealing for long term storage (>1 mo).

### Preparation of DBP

Demineralized bone pieces were first cut for their height to fit the diameter of the mounting disc (3-4 cm) and for their length to be less than or equal to the size of the blade (8 cm). The trimmed bone piece was embedded in an optimal cutting temperature medium parallel to the mounting disc and frozen at −20 C°. The embedded bone piece was sliced into 20-μm thickness using a cryostat (CryoStar NX70) to generate vDBP. Separately, the bone piece was embedded in an optimal cutting temperature medium perpendicular to the mounting disc and frozen to generate 20-μm-thick tDBP. After the first few rounds of cutting, vertical and transversal collagen structures were confirmed using transmission light microscopy. DBPs were placed in 50-mL centrifuge tubes with 1% sodium dodecyl sulfate solution under gentle shaking for 1 h to remove the remaining cellular components and confirmed by nuclear DAPI staining. Decellularized DBPs were then washed with DI water 20 times and stored in 70% ethanol at 4 °C until use. Prior to cell culture, DBPs were biopsy-punched into 6-mm diameter circles. Inside a biosafety cabinet, the biopsy-punched DBPs were washed 5 times with 70% ethanol and 3 times with 1× phosphate-buffered saline (PBS), then placed in a 96-well plate and air-dried for stable adhesion to tissue culture plastic (TCP).

### Characterization of DBP

#### Optical transparency

DBPs were placed in a 96-well plate and UV-absorbance at 410 nm was measured for both empty wells and wells containing DBPs using a microplate reader (Synergy 2, BioTek). The absorbance data were then converted into UV transmittance values. The relative transmittance % was normalized by setting the transmittance of the empty wells as 100%.

#### Collagen structures

DBPs placed in a 96-well plate were scanned under a brightfield microscope with a 10× objective lens (EVOS). Obtained images were stitched to generate an entire DBP image. These images confirmed a uniaxially aligned collagen structure of vDBP and a concentric ring collagen structure of tDBP. Collagen fiber alignment angles were manually measured with the angle tool function in ImageJ using 10 measurements from 4 different samples. For vDBPs, the alignment angles were measured by drawing lines along each fiber and measuring their angles relative to the horizontal axis. For tDBPs, the angles between radii from a central point and tangent intersections were measured.

#### Molecular integrity

Structural integrity of triple helix collagen fibers was characterized in 2 different methods. First, resonant scanning multiphoton microscopy with a 25× objective lens (Nikon A1MP) was used to visualize collagen fibers on vDBP and tDBP via second harmonic generation (SHG), excited at 810 nm. Second, a 5-FAM-conjugated collagen hybridizing peptide (CHP) (3Helix) that binds to denatured collagen triple helices was used to assess the biochemical integrity of collagen fibers in vDBP and tDBP. DBPs treated in an 80 °C water bath for 1 min served as a positive control. Prior to use, the trimeric CHP stock solution was briefly immersed in the 80 °C water bath to dissociate into monomers thermally. The heated CHP solution was then rapidly cooled on ice for 30 s to prevent unnecessary thermal damage to the DBP samples. Subsequently, the prepared CHP solution was diluted to a low concentration (10 μM) and applied to both heat-denatured and untreated vDBP and tDBP samples. Following overnight incubation at 4 °C, we imaged the CHP-bound on the heat-denatured and untreated DBPs using a fluorescence microscope (EVOS).

#### Vascular regions

vDBPs and tDBPs were placed in a 96-well plate and imaged using brightfield microscope with a 10× objective lens. The images were stitched together to form a complete image of each DBP. The surface area covered by vascular regions was measured using Image J.

#### Isolation and expansion of primary murine osteogenic cells

DsRed mice were obtained from Dr B. Osborne (UMass-Amherst). B6 mice (000664) were obtained from Jackson Lab. A total of 3 male and female mice aged 4-10 wk were randomly selected and used. After CO_2_ euthanasia, femurs and tibias were extracted, and the connective tissues were removed. The epiphyses of the cleaned bone were cut and placed in 0.5-mL microcentrifuge tubes with a hole made in the bottom. This 0.5-mL tube was then placed inside a 1.5-mL tube and centrifuged at 10 000 g for 30 s to flush the BM. Next, the bones were placed in a Petri dish with 1 mL of PBS and cut into small pieces of 1-2 mm in length using a scalpel (size 21). These bone chips were incubated with α-minimum essential medium (α-MEM) supplemented with 10% fetal bovine serum (FBS), 1% penicillin–streptomycin (PS), and 800 U of collagenase at 37 °C and 5% CO_2_. After overnight incubation, the medium was replaced with α-MEM containing 1% PS and 10% FBS (expansion medium). Osteogenic cells migrated out from the bone chips including osteoblasts and a fraction of monocytes were expanded on TCP. Cells at passage 4 or less were used in experiments to avoid possible de-differentiation.

#### Osteoblast differentiation and characterization

Differentiation medium is prepared by adding β-glycerophosphate (10 mM) and L-ascorbic acid (200 μM) in the expansion medium. Osteoblasts in this manuscript indicate osteogenic cells that are cultured in the differentiation medium. Sterile vDBPs and tDBPs were placed into the wells of a 96-well plate. Random rat-tail collagen (RTC)-coated wells were prepared by adding 100 μL of collagen solution, following the protocol in the product manual. DsRed osteogenic cells were seeded on TCP, RTC, vDBP, and tDBP-placed well (1000 cells/well) and cultured for 1 wk while conducting fluorescence and brightfield imaging every 2 d. Upon reaching full confluence, cells were cultured in the osteogenic differentiation medium for an additional 2 wk. At culture termination, osteoblasts were fixed using paraformaldehyde (4%), stained with Alexa Fluor 488 phalloidin for cytoskeletal actin filaments and DAPI for cell nuclei, and observed using a fluorescence microscope. Initial cell adhesion angles after 24 h of seeding on substrates were measured using the angle tool function from ImageJ by manually identifying 3 points from a horizontal line (virtual) and an actin filament line (phalloidin stain). Subsequent cell proliferation was determined by measuring the percentage of red fluorescent surface area covered by DsRed osteoblasts over time using ImageJ.

### Acellular mineralization using SBF

#### SBF preparation

The 10× SBF was prepared using a standardized protocol reported.^23^ The final solution contained the following reagents: NaCl (1 M), KCl (5 mM), CaCl_2_·2H_2_O (25 mM), MgCl_2_·6H_2_O (5 mM), Na_2_HPO_4_ (10 mM), and the pH was maintained at 3.7 during dissolution by adding a few drops of 1.2 N hydrochloric acid solution, which was monitored with a pH meter. The final volume was adjusted to 1 L by adding DI water while increasing the pH to 4.

#### SBF mineralization

After preparing RTC, vDBP, and tDBP in a 96-well plate, 50 μL of NaHCO_3_ (50 mM) was added in each well and incubated for 15 min at 37 °C. Subsequently, 150 μL of 10× SBF solution was added and mixed with the NaHCO_3_ solution by pipetting to adjust the pH and initiate mineralization. Following 1 h incubation at 37 °C, the wells were washed 3 times with DI water. This process constituted 1 cycle, which was repeated for up to 3 cycles.

### Characterization of mineral deposition

#### Alizarin red mineral staining

For osteoblast mineralization, samples were first fixed with paraformaldehyde (4%) and stained with 20 mM alizarin red S (Catalog #: 400481000) for 30 min at room temperature. Samples were then washed with DI water repeatedly until the wash solution remained clear, indicating the removal of excess staining solution. Alizarin red stained samples were first imaged with an optical microscope (EVOS) with a 10× objective lens and then solubilized by adding acetic acid solution (50%) for 1 h. The acetic acid solution with solubilized alizarin red was transferred to a new well, and UV absorbance at 405 nm was measured with a microplate reader (BioTek). The same alizarin red staining and quantification procedures were performed without fixation for acellular mineralization in SBF.

#### Fluorochrome calcein mineral staining

Osteoblasts were cultured on vDBPs and tDBPs for 2 wk using an osteogenic differentiation medium. Every 2 days of culture, cells were washed with 1× PBS without calcium and magnesium ions and stained with 5-μM fluorochrome calcein (Catalog #: 0219508705) solution for 30 min to characterize mineral deposition. After 30 min, samples were washed with 1× PBS 3 times, and osteogenic differentiation medium was added for further culture. The calcein-stained DBPs and DsRed osteoblasts were imaged under a fluorescent microscope with a 10× objective lens. The dimensions and fluorescence intensity of calcein bound to mineral crystals were quantified with ImageJ.

#### Scanning electron microscope for morphological analysis of minerals

Re-mineralized vDBPs and tDBPs were heated at 500 °C using a furnace for 5 h to decompose the organic collagen fibers thermally. (Note: Heat treatment may possibly modify mineral growth, maturation, and the interface between collagen and mineral.) Fully decomposed vDBPs and tDBPs were then transferred to glass slides to image under optical microscopy and visualize the distribution of remaining mineral layers on each collagen fiber alignment. The samples were moved to a stub and coated with gold using a sputter coating machine (Cressington Sputter Coater 108). Scanning electron microscopy (FBI Magellan) was used to characterize the distribution of mineral layers.

#### Scanning electron microscope-energy dispersive X-ray spectroscopy for elemental analysis of minerals

Elemental analysis of minerals was conducted using scanning electron microscope-energy dispersive X-ray spectroscopy (SEM–EDX) (Apreo VolumeScope) on uncoated samples to analyze the elemental composition of the mineral layers on thermally treated (500 °C for 5 h) bovine compact bone discs, vDBPs, and tDBPs, with a focus on the mineral content in both bone and vascular regions.

### Characterization of osteoclast resorbing activity

#### Isolation of murine BMMs

The femur and tibia bones were collected from DsRed mouse. BM cells were obtained from the femur via centrifugation and plated on a 10-cm petri dish with α-MEM supplemented with 10% FBS and 1& P/S and incubated at 37 °C overnight to separate adherent stromal cells. The floating BMMs are then collected for osteoblast–osteoclast co-culture experiments.

#### Co-culture of osteoblast and osteoclast

Osteoblasts (10 000 cells/well) were seeded and cultured on vDBP and tDBP with osteogenic medium for 1 wk. Then, BMMs (1 000 000 cells/well) were introduced into the wells with a stimulation medium composed of α-MEM, 10% FBS, VD3 (10 nM), and PGE2 (1 μM), and monitored over time using a fluorescence microscope.

#### Osteoclast differentiation and characterization

Osteoclasts were fixed in paraformaldehyde (4%) and rinsed with PBS 3 times. Osteoclast differentiation was determined by examining multinucleation and tartrate-resistant acid phosphatase (TRAP) activity using nucleus DAPI staining and a TRAP detection kit (387A, Invitrogen), per the manufacturer’s protocol. Stained cells were then analyzed using fluorescence and optical microscopy. The size of multinucleated osteoclasts and the number of TRAP-stained osteoclasts were manually measured in Image J.

#### Quantitative analysis of osteoclast mineral resorption

Resorption areas and patterns were imaged every 24 h using a brightfield microscope with a 10× objective lens. Time-course resorbed areas and patterns were manually tracked and quantified in Image J. The percentage of the surface area resorbed on vDBP and tDBP was first measured, and the resorption rate was calculated by plotting the percentage of the resorbed area over time. An algorithm was established to differentiate between 2 types of resorption patterns observed in osteoclast activity: trenches and pits. Trenches are defined as larger, elongated areas of mineral resorption where the endpoint resorbed surface area exceeds twice the initial resorbed surface area measured at the start of the observation period. Pits are defined as smaller, rounded, hollow resorbed areas where the increase in resorbed surface area from the start to the endpoint is less than double. The measurements were carried out by tracking the same 25 areas across time-course images. This tracking was performed indecently for each of the 3 replicate samples.

#### Statistical analysis

All measurements were collected at least triplicate and expressed as means ± standard deviation. *p*-values were assessed by 2-way analysis of variance (ANOVA) when more than 3 groups were compared or 2-tailed Student’s *t*-test when 2 groups were compared. *p*-values and statistical analysis methods used are denoted in the figures. A *p*-value of less than .05 was deemed to indicate statistical significance. All quantitative data are presented as box plots representing the median (central line), interquartile range (box), and full data range (whiskers) with individual data points.

## Results

### Vertical and transversal DBPs present distinct collagen matrix structures

**Figure 1:**
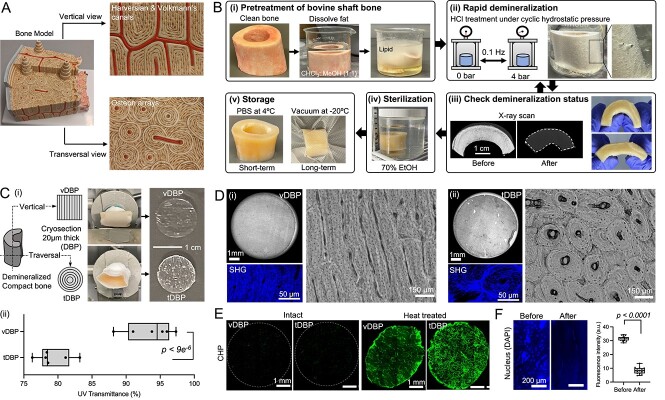
Preparation and characterization of vertical and transversal DBPs. (A) A bone model displays parallelly aligned osteon arrays with distinct lamellar structures of bony extracellular matrix from top and side views. (B) Procedure for preparing demineralized bovine compact bone. (i-ii) A femoral shaft bone was cut into ~5 cm pieces, dissolved fat, and demineralized in hydrochloric acid under cyclic hydrostatic pressure. (iii) Demineralization was confirmed by manual bending, and full demineralization was validated by X-ray transparency. (iv) Demineralized compact bone is sterilized in 70% ethanol. For long-term (>1 mo) storage, it was vacuum sealed and placed at −20 °C. (C) Procedure for preparing DBP. (i) The demineralized bone piece was mounted on a cold stage in either vertical or transversal direction with optimal cutting temperature medium and cryo-sectioned into 20-μm thickness. A large DBP (>3 × 3 cm) was then trimmed into a circular shape using a biopsy punch (D = 6 mm) to be attached to a 96-well plate. vDBP and tDBP exhibit distinct light reflections. (ii) Comparison of UV transmittance between vDBP and tDBP relative to TCP. (*n* = 6 independent samples). (D) Representative optical microscopy images of (i) vDBP that shows a linearly aligned lamellar structure of collagen matrix and (ii) tDBP that shows a concentric circular lamellar structure of collagen matrix. Multiphoton SHG images show the distinct organization of collagen fibers between vDBP and tDBP (bottom left). (E) Confirm intact structural integrity of triple helix collagen fibrils using CHP conjugated with fluorescent dye. Heat denatured vDBP and tDBP were used as positive controls. Representative images from 3 independent experiments. (F) Confirm removal of residual cellular materials after sodium dodecyl sulfate treatment by nuclei DAPI staining (*n* = 15). Representative images from 3 independent experiments. Statistical analysis was performed using Student’s *t*-test. Abbreviation: A.U., arbitrary units.

In large vertebrates, compact bone mainly consists of vertically aligned osteons, the fundamental structural units that confer strong directional mechanical support. These osteons display concentric collagen layers around central blood vessels when viewed from the top. In contrast, a side-view reveals aligned collagen structures, including Volkmann’s and Haversian canals for blood vessel networks (Figure 1A). We hypothesized that the role of collagen structure in regulating mineral metabolism could be substantiated using vertical and transversal DBP sections that exhibit distinct collagen structures while preserving natural bony collagen complexity. To test the hypothesis, we first demineralized bovine femoral shaft bone with hydrochloric acid (1.2 N) under cyclic compressed air (4 bars, 0.1 Hz). Repeated monitoring of the demineralization process through manual bending and X-ray scanning confirmed the complete removal of mineral content, as evidenced by the increased flexibility of the bone and the absence of mineral in X-ray imaging (Figure 1B).

Next, we prepared DBP by cryo-sectioning demineralized bone into 20-μm-thin sections in vertical and transversal directions. One notable difference between tDBP and vDBP was in light reflection. The UV transmittance of vDBP relative to TCP was 93.6 ± 3.4% compared with 7.3 ± 2.5% for tDBP (Figure 1C). Both DBPs retained mechanical durability and semi-transparency that facilitates easy handling and observing collagen patterns. vDBP and tDBP adhered firmly to TCP after overnight air-drying. vDBP showed parallel lamellae structures with blood network canals, whereas tDBP displayed densely packed osteons and concentric lamellae around central blood vessels. Using second-harmonic generation multiphoton microscopy, we confirmed the triple-helix structure of collagen fibers in DBP (Figure 1D). The structural integrity of collagen within DBPs was confirmed using a fluorescent dye-conjugated collagen hybridizing peptide, which selectively binds to denatured collagen fibers. No fluorescence indicates intact collagen (Figure 1E). Finally, treating DBPs with 1% sodium dodecyl sulfate removed residual cellular materials, confirmed by significantly reduced nucleus DAPI staining (Figure 1F). These results confirm that DBP preserves both the structural integrity and the organization of the collagen matrix in natural bone. The contrasting collagen patterns in vDBP and tDBP provide a valuable framework for substantiating how these patterns affect the mineralization process in bone.

### DBPs promote SBF-based acellular mineralization independent of collagen patterns

**Figure 2:**
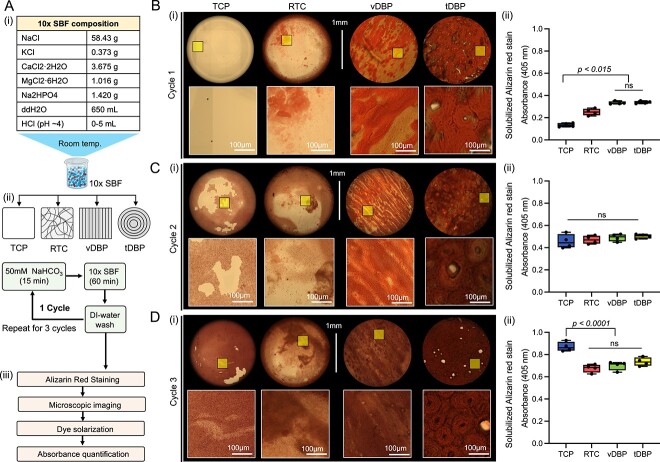
Comparison of SBF-based acellular mineralization of different collagen structures. (A) (i) Composition of 10× SBF, (ii) schematic of SBF-based acellular mineralization of TCP, RTC, vDBP, and tDBP. Each mineralization cycle consists of 15 min in NaHCO_3_ (50 mM), followed by 60 min in 10× SBF, and final rinsing with DI water. TCP and randomly coated rat-tail collagen-coated TCP (ie RTC) were used as controls. (iii) The mineral deposition was visualized and quantified by alizarin red staining. (B–D) (i) Microscopy scanning of alizarin red-stained TCP, RTC, vDBP, and tDBP and (ii) quantified mineral deposition by measuring UV absorbance of solubilized alizarin dye stain at 405 nm. (B) Cycle 1, (C) cycle 2, and (D) cycle 3. (*n* = 4 independent samples). Statistical analysis was performed using 2-way analysis of variance (ANOVA); ns = not significant (*p*>.05).

We examined the impact of distinct collagen structures of vDBP and tDBP on regulating acellular mineralization using SBF, which mimics the ionic composition of human blood plasma. TCP was used as a negative control, while random rat-tail collagen (RTC)-coated TCP served as a nonstructural collagen control. The acellular mineralization cycle was repeated 3 times to promote a sufficient mineral layer formation across all substrates—TCP, RTC, vDBP, and tDBP. Deposited mineral was visualized and quantified using alizarin red staining (Figure 2A). In the first cycle, collagen-presenting wells (RTC, DBPs) exhibited significantly greater mineralization than TCP. DBPs supported notably more mineral deposition than RTC, but there was no significant difference between vDBP and tDBP (Figure 2B). In the second cycle, mineral deposition increased, and quantified minerals were comparable across all groups. However, on TCP and RTC that have no and low collagen, mineral deposition was less stable than DBPs, which made them prone to detachment during rinsing (Figure 2C). In the third cycle, mineral deposition further increased in all groups, with the most significant increase on TCP. Quantified mineral deposition among RTC, vDBP, and tDBP was comparable (Figure 2D). These results affirm that a pre-existing collagen matrix facilitates mineral deposition, but the distinct collagen structures of tDBP and vDBP showed no significant difference in acellular mineralization in 10× SBF.

### DBP collagen structures direct osteoblast adhesion and proliferation

**Figure 3:**
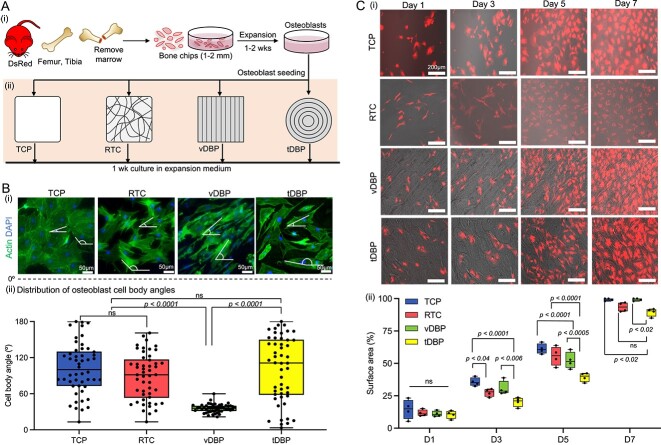
Comparative murine osteoblast adhesion and proliferation on different collagen structures. (A) (i) Experimental schematic and timeline for isolating murine osteoblasts from DsRed reporter mice and expanding them. (ii) Comparative osteoblast culture on TCP, RTC, vDBP, and tDBP to determine the impact of collagen structures in directing osteoblast adhesion and proliferation. (B) (i) Representative morphology of osteoblasts after 1 wk of culture from 3 independent experiments. (Actin filament and nuclei were stained with phalloidin and DAPI, respectively). (ii) Distribution of osteoblast cell body alignment angles (*n* = 50 individual cells). (C) (i) Representative time-course fluorescent monitoring of osteoblast proliferation on TCP, RTC, vDBP, and tDBP for 1 wk from 3 independent experiments. (ii) Comparative measurement of DsRed osteoblast-covered surface area on day 1, 3, 5, and 7 (*n* = 4 independent samples). Statistical analysis was performed using 2-way analysis of variance (ANOVA); ns = not significant (*p*>.05).

We investigated the role of DBP collagen structures in directing osteoblast adhesion and growth. While DBP is translucent, identifying individual cells under microscopy imaging is difficult due to the underlying collagen structure. To overcome this issue, we used genetically labeled primary osteoblasts from DsRed reporter mice. Osteogenic cells retrieved from femoral and tibial bone chips were seeded on TCP, RTC, vDBP, and tDBP placed in a 96-well plate. After 1 wk of culture in osteogenic differentiation medium, we fixed the osteoblasts and conducted actin filament and nucleus staining (Figure 3A). Osteoblasts exhibited distinct morphology on each substrate. On vDBP, osteoblasts displayed a spindle shape with a directionally elongated morphology. In contrast, on tDBP and TCP, they exhibited a more spread morphology with less directional alignment. On RTC, osteoblasts display a more random and less ordered morphology in response to the randomly oriented collagen fibers. Characterized cell body alignment angles on vDBP and tDBP were 35.7 ± 6.9° and 101.9 ± 53.5°, respectively (Figure 3B).

DsRed reporter osteoblasts allowed for longitudinal quantitative fluorescent monitoring of cellular adhesion and growth on DBPs. Osteoblasts adhered to DBP by following the underlying collagen patterns. On vDBP, osteoblasts landed on linearly aligned collagen fibers and displayed an elongated morphology. On tDBP, osteoblasts adapted to circumferentially aligned collagen fibers showed a bent morphology. These initial adhesion patterns are maintained during proliferation, resulting in distinct patterns between vDBP and tDBP. Interestingly, osteoblasts preferentially adhered to vascular regions. On RTC, osteoblasts initially showed more elongated morphology, but as they proliferated and reached confluence, their morphology became similar to osteoblasts on TCP. Quantitative analysis of the surface-covered osteoblast area confirmed that osteoblasts on tDBP proliferated significantly slower than TCP, RTC, and vDBP (Figure 3C). These results underscore the critical role of collagen structures in regulating osteoblast adhesion and growth.

### DBP collagen structures affect the rate of osteoblastic mineral deposition

**Figure 4:**
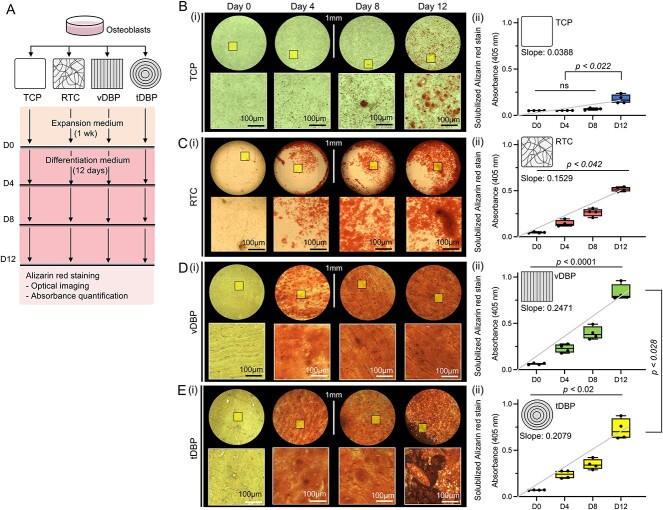
Comparative osteoblastic mineralization on different collagen structures. (A) Experimental schematic and timeline to determine the role of collagen structures in directing osteoblastic mineralization on TCP, RTC, vDBP, and tDBP. (B–E) (i) Representative images of alizarin dye stained osteoblastic mineralization and (ii) quantifications of solubilized alizarin dye on (B) TCP, (C) RTC, (D) vDBP, and (E) tDBP at day 0, 4, 8, and 12. The slope of mineral deposition was calculated using the mean value of each time point (*n* = 4 independent experiments in each time point). Statistical analysis was performed using 2-way analysis of variance (ANOVA); ns = not significant (*p*>.05).

We hypothesized that DBP collagen structures regulate the rate of osteoblastic mineral deposition. To test this hypothesis, we first seeded osteoblasts on TCP, RTC, vDBP, and tDBP and cultured them in an expansion medium for 1 wk to reach confluence. We then switched to an osteogenic differentiation medium and cultured for 12 days while quantitatively measuring deposited minerals using alizarin red staining on days 0, 4, 8, and 12 (Figure 4A). On TCP, evident mineralization in the form of small nodules took at least 1 wk. Quantitative analysis of alizarin red staining confirmed no significant mineral deposition by day 8. Increased mineral deposition was detected on days 8 and 12 but remained modest (Figure 4B). On RTC, pre-existing collagen structures supported faster mineralization than TCP. Quantitative analysis confirmed significantly higher mineral deposition after day 4 (Figure 4C). Osteoblastic mineral deposition was markedly faster on DBPs, irrespective of vDBP and tDBP. On day 0, a modest but statistically significant alizarin red signal was detected on DBPs compared with TCP, indicating spontaneous mineral deposition by osteogenic cells during the 1-wk culture period in the expansion medium. Substantial mineral deposition was consistently observed across the entire surface of DBPs and persisted throughout the 12-d culture period, gradually reducing the optical transparency (Figure 4D). Quantitative analysis of mineral deposition over time confirmed significantly faster and continued mineralization for 12 days. For a quantitative comparison of mineral increase rates, we derived a linear trend line, from which slope was derived. The rate of osteoblast mineral deposition, as characterized by the slope of increasing solubilized alizarin red absorbance measurement over 12 d, was higher on vDBP (0.25) than tDBP (0.21) (Figure 4E). These results confirmed that DBPs preserving natural collagen structure of bone support rapid and substantial osteoblastic mineral deposition. At the same time, the collagen structures of DBPs influence the kinetics of osteoblastic mineralization.

### DBP collagen structures affect the spatiotemporal osteoblastic mineral deposition

**Figure 5:**
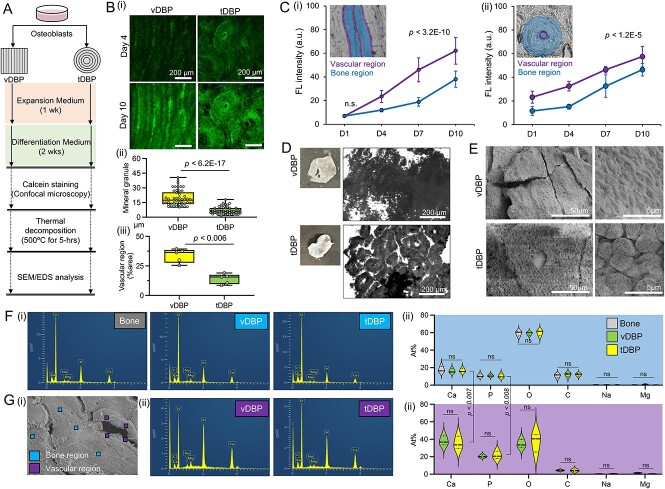
Collagen structures and anatomical locations of DBPs direct osteoblastic mineralization. (A) Experimental schematic and timeline. (B) (i) Representative time-course fluorescent calcein stained images of osteoblastic mineral deposition on vDBP and tDBP, (ii) measurement of mineral granules size after 2 wk of culture (*n* = 50 measurements from 3 independent experiments), and (iii) comparison of vascular regions between vDBP and tDBP (*n* = 5 independent samples). (C) Time-course measurement of osteoblastic mineralization between vascular and bone regions by quantifying fluorescent calcein intensity in (i) vDBP and (ii) tDBP (*n* = 21 measurements from 3 independent samples). Representative delineation of vascular and bone regions in vDBP and tDBP. (D) Representative images of deposited mineral on vDBP and tDBP after thermal decomposition from 3 independent experiments. Gross camera images (left) and optical microscopy images (right). (E) Representative SEM images of deposited mineral on vDBP and tDBP from 3 independent experiments. (F, G) SEM–EDX analysis of thermally decomposed (F) bovine compact bone and bone regions of vDBP and tDBP, and (G) (i) vascular regions of vDBP and tDBP, (ii) representative EDX profile, and (iii) quantified elements (*n* = 3 independent measurements). Statistical analysis of element compositions in bone regions was performed using 2-way analysis of variance (ANOVA). Statistical comparison of calcium and phosphate between vascular and bone regions was performed using Student’s *t*-test; ns = not significant (*p*>.05).

We next comparatively investigated the osteoblastic mineral deposition between vDBP and tDBP, under the hypothesis that collagen structures of DBP direct mineralization patterns. After 1 wk of culture on DBPs in an expansion medium, we switched to an osteogenic differentiation medium and monitored mineralization for 2 wk (Figure 5A). Time-course fluorescent calcein staining and confocal microscopy imaging visualized the mineral deposition processes following the underlying collagen structures of DBP. On tDBP, deposited minerals displayed a concentric circumferential organization, whereas on vDBP, minerals were deposited in a parallelly aligned manner. Characterized size of mineral granules after 2 wk of culture on vDBP was 3-fold larger than tDBP. Interestingly, vascular regions exhibited higher mineral deposition than bone regions regardless of vDBP and tDBP. Vascular regions were significantly more prevalent in vDBP than in tDBP (Figure 5B). Spatiotemporal monitoring of the mineralization process confirmed that vascular regions exhibited faster mineralization than bone regions regardless of vDBP and tDBP. On vDBP, the difference between these vascular and bone regions gradually increased over time, whereas on tDBP, the difference was persistent during the culture period (Figure 5C).

Next, we thermally decomposed tDBP and vDBP at 500 °C for 5 h, which removed organic components and showed the remaining minerals. One notable gross observation was that thermally decomposed vDBP partially rolled, whereas tDBP consistently maintained a flat structure. Although heat treatment has the potential to modify mineral maturation and growth, optical microscopy imaging confirmed that the deposited minerals followed the collagen pattern (Figure 5D). We further conducted SEM images to characterize mineral structures between vDBP and tDBP comparatively. On vDBP, where mineralization occurred following the linearly aligned collagen fibers, minerals exhibited a small and integrated granular morphology. On tDBP, which directs mineralization following the concentric collagen pattern, minerals showed a more prominent and less integrated granular shape (Figure 5E). This suggests that the underlying collagen structure continues to guide mineral deposition despite potential modifications from the heat treatment.

Finally, we conducted an elementary analysis of deposited mineral using SEM-EDX, with thermally decomposed bovine compact bone disc as a control. SEM-EDX analysis was performed for vascular and bone regions. The profiles of SEM-EDX analysis showed comparable peaks across vascular and bone regions of vDBP and tDBP. Characterized calcium and phosphonate ratios were 1.64 for the bone, 1.47 for vDBP, and 1.60 for tDBP (Figure 5F). We then comparably characterized SEM-EDX analysis between vascular and bone regions in vDBP and tDBP. As expected, SEM-EDX analysis of calcium and phosphate compositions was significantly higher in vascular regions compared with bone regions regardless. The characteristic calcium and phosphonate ratio in vascular regions of vDBP was 1.86, and tDBP was 1.64. However, SEM-EDX analysis did not capture other elements in vascular regions (Figure 5G). Collectively, these results underscore the role of collagen structures and local extracellular matrix compositions in directing osteoblast mineral deposition.

### DBP collagen structures affect osteoclast resorption activity and patterns

**Figure 6:**
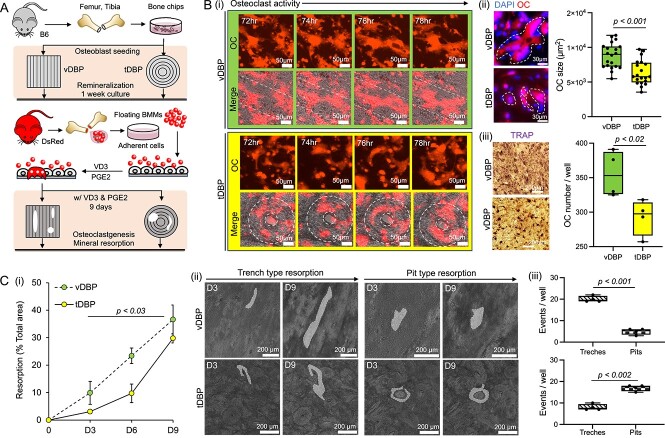
Comparative osteoclast activity and mineral resorption patterns between vDBP and tDBP. (A) Schematic of experimental setup and procedures. Osteoblasts derived from B6 mice were cultured on DBPs for 1 wk to re-mineralize DBPs. BMMs derived from DsRed mice were introduced. The co-culture was stimulated with vitamin D3 (VD3) and prostaglandin E2 (PGE2) for 9 d to induce osteoclast differentiation. Time-course osteoclast differentiation and their mineral resorption patterns were monitored under fluorescent and brightfield microscopy imaging, respectively. (B) (i) Representative time-course fluorescent microscopy images of mature osteoclasts on vDBP and tDBP. (ii) Representative images of osteoclasts on vDBP and tDBP confirmed by a DsRed cell including multiple nuclei (DAPI) and comparison of osteoclast size (*n* = 20 osteoclasts from 4 independent samples). (iii) Representative TRAP-stained images on vDBP and tDBP and quantified osteoclast number per well (*n* = 4 independent samples). (C) (i) Time-course quantification of osteoclastic mineral resorption area between vDBP and tDBP (*n* = 3 independent samples). (ii) Representative time-course images of trench- and pit-type resorption on vDBP and tDBP from day 3 to day 9. White outlines indicate resorption areas by osteoclasts. (iii) Quantitative comparison of trench- and pit-type resorption events between vDBP and tDBP (*n* = 25 from 4 independent samples). Statistical analysis was performed using Student’s *t*-test; ns = not significant (*p*>.05).

Bone resorption occurs when osteoclasts adhere to and degrade the mineralized collagen matrix. To determine whether the collagen alignment in DBP impacts the resorptive phase of bone remodeling, similarly to its effects on osteoblastic activity, we differentiated BMMs into osteoclasts in co-culture with osteoblast. Osteoblasts derived from B6 mice (nonfluorescent) were cultured on vDBP and tDBP for 1 wk to remineralize the surface. BMMs retrieved from DsRed mice were then introduced with VD3 (10 nM) and PGE2 (1 μM) to stimulate osteoblasts. In previous studies, VD3/PGE2 stimulated osteoblasts on DBP shift their secretion profiles of osteoprotegerin and RANKL, which induces osteoclastogenesis of co-cultured BMMs^21^^,^^22^ (Figure 6A). As expected, VD3/PGE2-stimulated osteoblasts led to the differentiation of BMMs into multinucleated osteoclasts, confirmed by multinuclei and TRAP staining. On vDBP, osteoclasts displayed an elongated morphology, aligning along the direction of the collagen fibers. On tDBP, osteoclasts appeared more rounded with fewer protrusions, consistent with the nonlinear, concentric collagen alignment. Characterized size (multinuclei DsRed) and resorption activity (TRAP) of osteoclasts were notably higher in vDBP than in tDBP (Figure 6B). These results indicate that osteoclasts are responsive to collagen structures, which could also affect their mineral resorption activity and patterns.

Next, we conducted a time-course comparative monitoring of mineral resorption between vDBP and tDBP by measuring the surface area of decreased brightness in the mineralized DBP. The imaging study confirmed that vDBP facilitated significantly higher resorption compared with tDBP (Figure 6C). This result aligns with the above observation, indicating that vDBP supports greater osteoclast activity than tDBP. Finally, we examined the effect of underlying mineralized collagen structures on directing osteoclast mineral resorption patterns, focusing on trench and pit types. Trench-type resorption is characterized by extended and linear mineral degradation along the collagen fibers, whereas pit-type resorption is characterized by localized and rounded intermittent resorption. Although vDBP and tDBP support both trench- and pit-type resorption, trenches were primarily observed on vDBP, while pits were more commonly identified (Figure 6C). Altogether, these results substantiate the impact of mineralized collagen structures on regulating osteoclastic mineral resorption patterns.

## Discussion

Mineral metabolism is a crucial physiological process for maintaining the mechanical structure^24^ and mineral balance of bones. Dysregulated bone metabolism can lead to various pathological conditions, including osteoporosis,^25^ osteomalacia,^26^ fibrodysplasia ossificans progressive,^1^ and bone metastasis.^27^^,^^28^ While the significance of the underlying collagen structures in guiding hydroxyapatite crystal nucleation and calcium phosphate mineral growth is documented, detailed investigation is required to unravel the mechanisms of these pathological conditions and develop better treatment strategies. However, this remains an intrinsic challenge due to the anatomical inaccessibility of the inner bone cavity in animal models. In vitro recapitulation of bone metabolism represents a great promise, but one major challenge is the lack of relevant and analytical biomaterial platforms that can replicate essential cellular and extracellular processes.^29-32^ DBP offers a unique opportunity to address this critical gap. By thin sectioning demineralized compact bone matrix in vertical and transversal directions, we obtained aligned and concentric collagen organization of DBP. Translucent DBP reveals these distinct collagen structures and allows longitudinal monitoring of cellular and extracellular processes under fluorescent and brightfield microscopy. DBP also offers robust analytical features for quantitatively assessing the extent and spatial distribution of mineral deposition and resorption. Previously, we reported functional and analytical replication of osteoblastic bone formation and osteoclastic bone resorption primarily on vDBP.^21^^,^^22^ In this study, we utilized both vDBP and tDBP to determine the functional connection between collagen structures and osteoblast and osteoclast-driven mineral metabolism.

SBF-mediated acellular mineralization underscores the role of the collagen matrix in regulating mineralization. vDBP, tDBP, and RTC exhibited higher initial mineralization compared with TCP, confirming the significance of pre-existing collagen matrix as a template for triggering nucleation and subsequent mineral growth.^33^ Repeated cycles of SBF exposure increased mineral deposition across all groups but with different increments. The fold-increase in mineral deposition after 3 cycles of SFB was 2.8 on RTC, whereas it was 2.1 on vDBP and 2.2 on tDBP. On TCP, mineralization occurred without collagen matrix, possibly because ionic spices in SBF are attracted to the charged surface where they nucleate mineral, which then grows in subsequent cycles. In the absence of collagen, these mineral layers are unstable and readily detached during liquid handling (Figure 2B). However, acellular mineralization was less sensitive to variations in collagen structures compared with osteoblastic mineralization as there was no significant difference between vDBP and tDBP. Reducing the kinetics of acellular mineralization, potentially by diluting SBF concentration or decreasing temperature, could substantiate the role of collagen structures in mineralization.

DBP collagen structures influence osteoblast adhesion and growth, indicating that osteoblasts detect the structural organization of collagen fibers, potentially through integrin-mediated focal adhesions.^34^ This interaction could trigger cytoskeletal rearrangement and subsequent cellular organization. Interestingly, osteoblasts preferentially adhered to regions where blood vessels exist and initiated mineralization faster than in the compact bone regions. Blood vessels within osteons comprise endothelial cells, osteoblasts, and macrophages with a more heterogeneous ECM composition, including type IV collagen and laminin.^35-37^ In contrast, compact bone regions mainly consist of type I collagen.^38^ SEM-EDX analysis confirmed that minerals deposited on DBPs by osteoblasts closely resembles the composition of hydroxyapatite in natural bone, where the calcium and phosphate ratio is maintained around 1.67^39^ (Figure 5F). SEM-EDX analysis also distinguished significantly more calcium and phosphate deposition in vascular regions but is limited to identifying trace ECM components (Figure 6F). These results implicate that ECM composition is also important in directing osteoblastic mineralization with collagen structure. While thermal decomposition of the collagen matrix facilitated mineral characterization, this process could mature mineral structures and induce their growth. Future research focusing on detailed characterization of localized ECM components in vascular regions, including collagen matrix, will provide insights into what causes increased osteoblast adhesion and mineralization.

In this study, we generated DBP from bovine femoral bone sourced from a slaughterhouse and local grocery stores. These bones processed at least 1 freeze and saw cycle, which may diminish bony collagen structures, although CHP-based characterization did not show denatured collagen fibrils. DBP can also be derived from other vertebrates, including human cadavers, porcine, bison, and chicken. Each type of vertebrate exhibits distinct macroscopic bone structures (eg, plexiform in bovine). Within the same vertebrate, bone structure differs depending on anatomical location (eg, femurs vs. skull).^40^ Nevertheless, fundamental collagen structures of bone that facilitate mineralization are evolutionarily conserved across vertebrates. Further investigation into potential collagen damage from freeze–thaw cycles and cold storage, as well as exploration of DBP derived from other vertebrate bones, coupled with detailed examination of collagen structure, will yield valuable insights for standardizing DBP production and implementing quality control measures for reproducible experimentation.

DBP holds great promise for studying the fundamental mechanisms of collagen mineralization and has significant translational potential in both research and clinical settings. For example, established osteoblast culture and mineralization on DBP can be harnessed to quantitatively measure the potential of various anabolic agents, including hormones (eg, estrogen, parathyroid hormones),^41^^,^^42^ growth factors (eg, bone morphogenic proteins, insulin growth factors),^43^^,^^44^ biomolecules (eg, vitamin D3, pyrophosphatase, calcium ions),^45-47^ and mechanical stimulation (eg, shear stress, vibration).^48^ Development of DBP-based bone anabolic assays will greatly accelerate bone anabolism-targeting drug discovery and development.

We employed remineralized vDBP and tDBP to investigate the effect of mineralized collagen structures on regulating osteoclastic bone resorption. Co-culturing osteoblasts and BMMs on DBP under stimulation with VD3 and PGE2 demonstrated robust osteoclastogenesis and mineral resorption.^21^ Osteoclasts also undergo fusion and fission, a newly identified osteoclast process in vivo.^49^ Trench and pit types of osteoclastic resorption have been reported in vivo and reproduced on bone discs.^20^ These osteoclastic resorption types were reproduced on DBP, but there were significantly different patterns depending on collagen structures. On vDBP, resorption is primarily a trench type, whereas on tDBP, resorption is mainly a pit type (Figure 6C). Previous studies have focused on osteoclasts’ intrinsic capability in conducting either type of mineral resorption,^50^ which may implicate osteoclast-driven bone metabolic disorders. Notably, our studies indicate that trench or pit types of osteoclast resorption may also be affected by underlying mineralized collagen structures. Future research can be extended to determine the correlation between osteoclast resorption type and the quality of bone structure as a potential diagnostic marker.

From a translational perspective, the current DBP-based bone model can be humanized by incorporating human osteoblasts, BM stromal cells, and precursors for osteoclasts. This refinement will significantly enhance the accuracy of the model and its ability to replicate human bone mineral metabolism. The enhanced predictive power of humanized DBP-based bone models will advance preclinical bone anabolic agent screening. Researchers will be able to identify and evaluate potential therapeutic agents with greater confidence and accuracy, accelerating the development of effective bone regeneration therapies. Advanced insights gained from DBP-based bone mineral metabolism studies will guide the development of improved biomaterials for clinical bone tissue regeneration. By understanding the intricate relationship between collagen structure, ECM composition, and bone mineralization, researchers can design biomaterials that effectively mimic the natural bone environment and promote osteogenesis and mineralization. Indeed, the significance of in vitro human tissue models as an alternative to preclinical animal studies has been formally recognized by the FDA Modernization Act 2.0.^51^ We envision that the DBP-based mineralized bone model will play a pivotal role in advancing these crucial research directions.

## Data Availability

The data will be available upon reasonable request.
